# Impact of the *AHI1* Gene on the Vulnerability to Schizophrenia: A Case-Control Association Study

**DOI:** 10.1371/journal.pone.0012254

**Published:** 2010-08-18

**Authors:** Olga Rivero, Andreas Reif, Julio Sanjuán, María D. Moltó, Sarah Kittel-Schneider, Carmen Nájera, Theresia Töpner, Klaus-Peter Lesch

**Affiliations:** 1 Unit of Molecular Psychiatry, Department of Psychiatry, Psychosomatics and Psychotherapy, University of Würzburg, Würzburg, Germany; 2 Department of Genetics, University of Valencia and Centre of Biomedical Research Network on Mental Health (CIBERSAM), Burjassot, Valencia, Spain; 3 Teaching Unit of Psychiatry, Department of Medicine, University of Valencia and Centre of Biomedical Research Network on Mental Health (CIBERSAM), Valencia, Spain; Innsbruck Medical University, Austria

## Abstract

**Background:**

The Abelson helper integration-1 (*AHI1*) gene is required for both cerebellar and cortical development in humans. While the accelerated evolution of *AHI1* in the human lineage indicates a role in cognitive (dys)function, a linkage scan in large pedigrees identified *AHI1* as a positional candidate for schizophrenia. To further investigate the contribution of *AHI1* to the susceptibility of schizophrenia, we evaluated the effect of *AHI1* variation on the vulnerability to psychosis in two samples from Spain and Germany.

**Methodology/Principal Findings:**

29 single-nucleotide polymorphisms (SNPs) located in a genomic region including the *AHI1* gene were genotyped in two samples from Spain (280 patients with psychotic disorders; 348 controls) and Germany (247 patients with schizophrenic disorders; 360 controls). Allelic, genotypic and haplotype frequencies were compared between cases and controls in both samples separately, as well as in the combined sample. The effect of genotype on several psychopathological measures (BPRS, KGV, PANSS) assessed in a Spanish subsample was also evaluated. We found several significant associations in the Spanish sample. Particularly, rs7750586 and rs911507, both located upstream of the *AHI1* coding region, were found to be associated with schizophrenia in the analysis of genotypic (p = 0.0033, and 0.031, respectively) and allelic frequencies (p = 0.001 in both cases). Moreover, several other risk and protective haplotypes were detected (0.006<p<0.036). Joint analysis also supported the association of rs7750586 and rs911507 with the risk for schizophrenia. The analysis of clinical measures also revealed an effect on symptom severity (minimum P value = 0.0037).

**Conclusions/Significance:**

Our data support, in agreement with previous reports, an effect of *AHI1* variation on the susceptibility to schizophrenia in central and southern European populations.

## Introduction

Because of its early onset, severity and chronic course schizophrenia is one of the most devastating neuropsychiatric disorders and represents a major public health concern [1]. Although the pathogenetic mechanisms leading to this disorder are largely unclear, it is well accepted that schizophrenia is the result of a complex interplay between genetic variants and environmental factors of different nature [2]–[4]. Until now, the search for susceptibility genes has given promising results, although some inconsistencies have also arisen [5]–[7]. Interestingly, several linkage studies have located putative schizophrenia as well as bipolar disorder susceptibility loci on the long arm of chromosome 6, and particularly in the 6q15–23.2 region [8]. Different genome-wide association studies (GWAS) also support association between Single Nucleotide Polymorphisms (SNPs) located in 6q21–6q25 and psychotic disorders [9]–[12]. In agreement with these findings, an autosomal scan of Arab-Israeli families [13], [14] supported linkage to schizophrenia to 6q23. This linkage peak contains a neurodevelopmental gene, the Abelson helper integration site 1 gene (*AHI1*). This gene is required for both cerebellar and cortical development in humans and several null-type and missense mutations in *AHI1* have been shown to cause Joubert syndrome, a rare autosomal recessive neurodevelopmental disorder, characterized by cognitive and behavioural disturbances among other clinical symptoms [15], [16].

The human *AHI1* gene contains 31 exons and generates at least three alternative isoforms. AHI1 protein, also known as Jouberin [16], contains seven Trp-Asp (WD) repeats, a Src homology 3 (SH3) domain and a coiled-coil domain. Jouberin is conserved in different mammalian species, particularly in the WD40 and SH3 domains [15]. The presence of both WD40 and SH3 domains in signaling and adaptor molecules suggests that *AHI1* may play an important role in signal transduction in normal cells, as an adaptor protein recruiting other signaling molecules and modulating and integrating their action [16], [17]. However, little is known about how *AHI1* is involved in the development of the central nervous system (CNS). *AHI1* mRNA is highly expressed in both the developing and the mature brain [15], [16]. Interestingly, it has been found [18] that murine Ahi1 protein forms a stable complex with huntingtin-associated protein 1 (Hap1). This complex appears to be critical for neonatal development through its function in intracellular trafficking, neurogenesis and neuronal differentiation.

Taken together, *AHI1* is an attractive candidate for a schizophrenia susceptibility gene and three association studies have been performed with promising results. In the first study in an inbred Arab-Israeli family sample and in an outbred nuclear family sample, Amann-Zalcenstein *et al.* [19] were able to identify seven markers strongly associated with schizophrenia. The association was found in a 500 kb linkage disequilibrium (LD) block on chromosome 6 harbouring *AHI1*. Subsequently, this association has been replicated in an independent European cohort from Iceland [20], in another large European sample [21], as well as in an enlarged sample from the original study [22]. In the latter, the significant SNPs were localized in a substructure of the large original LD block, which contains *AHI1* and the *C6orf217* gene, and may contain regulatory elements for both the *AHI1* and the neighboring phosphodiesterase 7B gene (*PDE7B*). Furthermore, there is also evidence of an association between an *AHI1* haplotype and autistic disorder (ASD) in a region of the gene that had been previously associated with schizophrenia [23].

Here we report the results from a case-control association study performed on two different samples from Germany and Spain. Our main aim was to test the hypothesis that *AHI1* variants were associated with schizophrenia as well as dimensional measures of disease severity.

## Results

### Spanish sample

SNP18 (rs4896156) deviated from Hardy-Weinberg Equilibrium (HWE) in the control sample (P = 3.89.10^−6^), clearly suggesting a low genotyping quality for this polymorphism. For this reason, this SNP was discarded.

**Figure 1:**
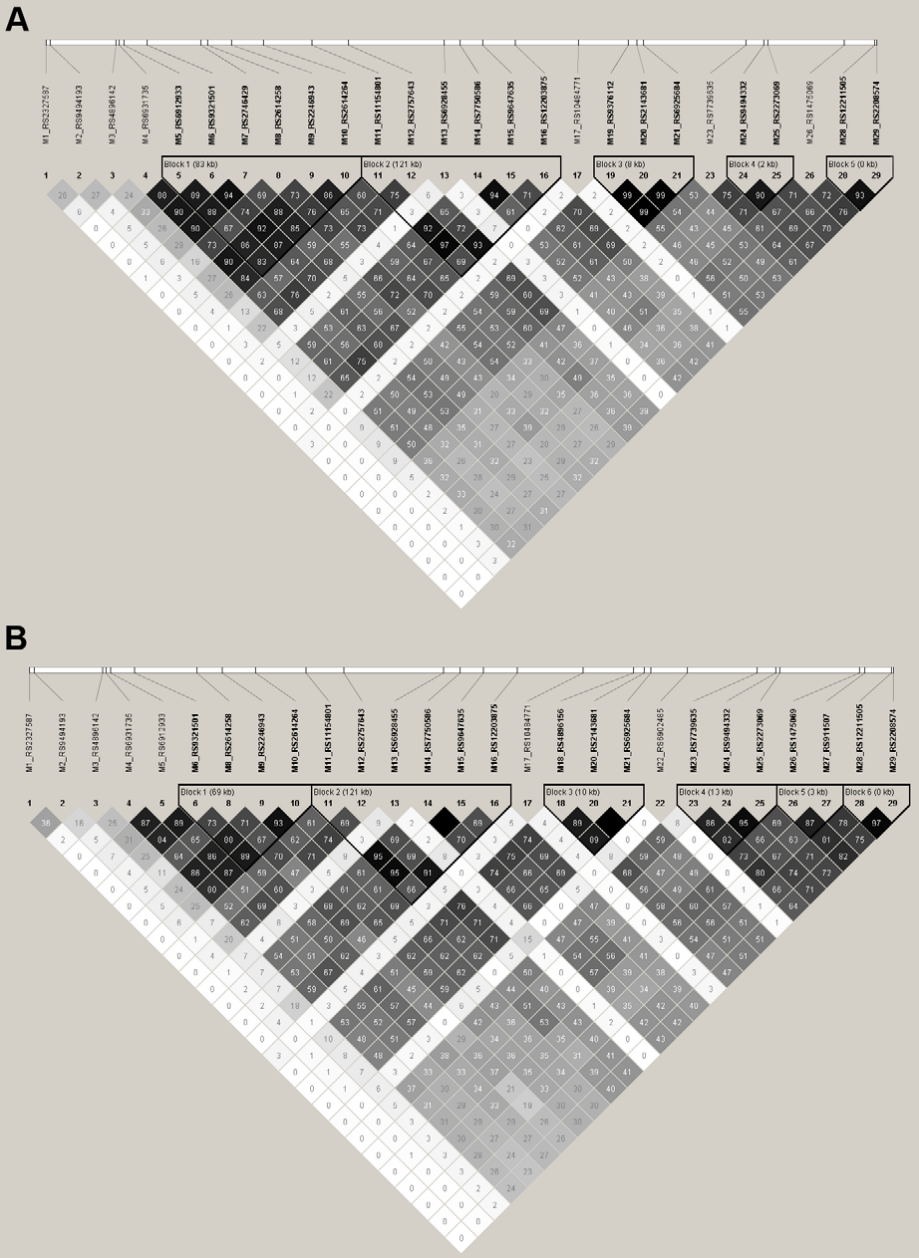
Linkage disequilibrium (LD) pattern of *AHI1* gene. Figures represent pairwise r^2^ values observed in control subjects from German (a) and Spanish (b) origin. Values are represented in a grayscale ranging from white (no LD) to black (high LD).

Figure 1a shows the pairwise linkage disequilibrium values observed in the control sample. The LD pattern from the schizophrenic sample (information not shown) was very similar. r^2^ values ranged between 0.0 and 0.993. According to Gabriel *et al.* [24] criteria, five LD blocks were detected. SNPs 1, 2, 3, 13 and 17 presented the lowest LD values (both r^2^ and D') when compared with the rest of the SNPs.

Single SNP association analysis revealed that SNP14 (rs7750586) was associated with psychosis, since both the G allele and the G/G genotype were more frequent in the group of patients with schizophrenia (P = 0.001 for the genotypic comparisons (recessive model); see table 1 for the allelic comparisons). The G allele of SNP27 (rs911507) also was associated with schizophrenia (table 1) and the same result was observed in the analysis of genotypic frequencies (P = 0.001, recessive model). Nevertheless, the significant values did not withstand the correction for multiple testing in the analysis of the allelic frequencies, although the *P* value corresponding to the analysis of genotypic frequencies still remained significant (P = 0.02 for both SNPs). We also performed a haplotype analysis, as well as an analysis of two-, three- and four marker haplotype sliding windows. As a result, several significant risk and protective haplotypes were detected (see table 1). The haplotypes encompassed SNPs 1, 2, 3 and 4 (located in the 3′ region of the analyzed genomic area), as well as markers 13, 14, 15 and 16 (located in intron 2 and in the 5′ putative regulatory region of *AHI1* gene). Interestingly, 5 of these significant haplotypes remained significant after a 1000-permutation run.

**Table 1:** Single alleles and haplotypes of the *AHI1* region associated with schizophrenia in the present study.

	GERMAN SAMPLE (N = 247 patients+360 controls)				SPANISH SAMPLE (N = 280 patients+348 controls)				COMBINED SAMPLE (N = 558 patients+708 controls)			
Single SNP or haplotype^a^	Cont freq	SCZ freq	OR (95% CI)	P	Cont freq	SCZ freq	OR (95% CI)	P	Cont freq	SCZ freq	OR (95% CI)	P
**SNP14 (rs7750586)**	0.353 (G)	0.323 (G)	0.88 (0.67–1.14)	0.315	0.286 (G)	0.380 (G)	1.53 (1.15–2.04)	**0.003 (0.086)**	0.325	0.360	1.52 (1.13–2.06)	**0.006**
**SNP27 (rs911507)**	0.342 (G)	0.356 (G)	1.07 (0.82–1.39)	0.619	0.370	0.441	1.34 (1.03–1.76)	**0.031 (0.81)**	0.331	0.386	1.34 (1.02–1.77)	**0.038** b
**SNP1-SNP4 (TAGG)**	0.108	0.081	0.43 (0.031–5.87)	0.277	0.0868	0.025	0.69 (0.19–3.91)	**0.006 (global:0.004)**	0.099	0.055	0.71 (0.19–2.54)	**0.011** (global:0.126)
**SNP1- SNP4 (TATA)**	0.222	0.223	0.57 (0.04–7.43)	0.853	0.212	0.305	3.41 (0.73–16)	**0.007 (ns after perm. run) (global:0.004)**	0.217	0.264	1.55 (0.45–5.29)	**0.027** (global:0.126)
**SNP13-SNP15 (TAA)**	0.588	0.600	0.85 (0.51–1.44)	0.663	0.628	0.555	0.91 (0.51–1.61)	**0.036 (global:0.044)**	0.603	0.578	0.89 (0.59–1.31)	0.324 (global:0.458)
**SNP13-SNP15 (TGC)**	0.348	0.328	0.79 (0.46–1.35)	0.469	0.293	0.380	1.33 (0.74–2.41)	**0.009 (global:0.044)**	0.325	0.353	1.00 (0.67–1.49)	0.255 (global:0.458)
**SNP14-SNP16 (AAA)**	0.572	0.588	1 (reference)	0.613	0.609	0.529	1 (ref)	**0.025 (ns after perm. run) (global:0.046)**	0.587	0.560	1 (ref)	0.276 (global:0.489)
**SNP14-SNP16 (GCG)**	0.345	0.328	0.92 (0.70–1.22)	0.584	0.288	0.369	1.48 (1.08–2.00)	**0.014 (global:0.046)**	0.321	0.348	1.13 (0.92–1.39)	0.237 (global:0.489)

Significant values are marked in bold. The corrected P-values are indicated in brackets.

a To avoid redundant information, other significant haplotypes, which are variations of other larger significant haplotypes from this table, have not been included.

b SNP27 had significantly different genotypic distributions in the German and the Spanish sample (heterogeneity *P* value = 0.016 uncorrected).

Abbreviations: Cont, control; SCZ, schizophrenia; ns, not significant; perm, permutation; ref, reference haplotype.

We next studied whether any of the SNPs associated to risk for psychosis in the single-SNP and haplotype analysis (SNP1, SNP2, SNP3, SNP4, SNP13, SNP14, SNP15, SNP16, and SNP27) had any impact on several clinical scores, namely Brief Psychiatric Rating Scale (BPRS), Psychiatric Assessment scale or KGV Symptom scale, and finally the total, general, positive and negative scores from the Positive and Negative Syndrome scale (PANSS). Significant results are displayed in table 2 with SNP4, SNP14 and SNP15 appearing to modulate the severity of psychotic symptoms, evaluated through PANSS, BPRS and KGV scales.

**Table 2:** *AHI1* SNPs significantly associated with clinical traits in the Spanish sample.

SNP	Genotypic distribution	Average response (SE)	Difference (95% CI)	Model	P^a^
**PANSS total score**					
**SNP4 (rs6931735)**	A/A–G/A = 61	61.98 (2.06)	0.00	Recessive	**0.05** (0.3)
	G/G = 19	70.68 (4.27)	**8.70 (0.12–17.29)**		
**SNP14 (rs7750586)**	A/A–A/G = 59	60.25 (1.66)	0.00	Recessive	**0.017** (0.136)
	G/G = 18	70.06 (4.85)	**9.80 (1.94–17.66)**		
**SNP15 (rs9647635)**	A/A–C/A = 60	63.58 (2.09)	0.00	Recessive	**0.023** (0.161)
	C/C = 13	75.62 (5.63)	**12.03 (1.86–22.20)**		
**PANSS General Score**					
**SNP4 (rs6931735)**	A/A–G/A = 61	31.54 (1.05)	0.00	Recessive	**0.042 (0.252)**
	G/G = 19	36.26 (2.37)	**4.72 (0.23–9.21)**		
**SNP14 (rs7750586)**	A/A–A/G = 59	30.78 (0.84)	0.00	Recessive	**0.016** (0.112)
	G/G = 18	36.17 (2.86)	**5.39 (1.11–9.66)**		
**SNP15 (rs9647635)**	A/A–C/A = 60	32.28 (1.02)	0.00	Recessive	**0.0037 (0.029)**
	C/C = 13	40.23 (3.25)	**7.95 (2.76–13.13)**		
**BPRS**					
**SNP14 (rs7750586)**	A/A–A/G = 59	44.8 (1.22)	0.00	Recessive	**0.045 (0.315)**
	G/G = 19	50.5 (3.15)	**5.70 (0.21–11.19)**		
**KGV scale**					
**SNP15 (rs9647635)**	A/A–C/A = 157	9.35 (0.41)	0.00	Recessive	**0.049** (0.392)
	C/C = 30	11.43 (1.05)	**2.08 (0.02**–**4.14)**		

Significant *P* values (P<0.05) are indicated in bold.

**^a^**The corrected *P* value is indicated in brackets.

Abbreviations: SE, standard error; CI, confidence interval.

### German sample

The genotyping for SNP7 and SNP19 did not pass the quality control in the sample of healthy subjects and were omitted from further analysis. LD structure for the control sample can be seen in figure 1b. These values were very similar for the affected subjects (data not shown) and ranged between 0.0 and 0.87. 6 blocks according to Gabriel *et al.* [24] criteria were detected. By contrast, the 3′ region of the *AHI1* gene (SNP1 to SNP3) featured lower pairwise LD values.

Regarding the single SNP association analysis (table 1), none of the 29 *AHI1* SNPs selected for the study differed in their allelic and genotypic frequencies between schizophrenic patients and controls (P<0.01). The haplotype analysis also yielded negative results.

### Combined sample

The German and the Spanish samples were combined to perform a heterogeneity test and an association analysis of the genotypic frequencies (Table 1) through Cochran-Mantel-Haenszel (CMH) tests, as well as a haplotype analysis through a retrospective likelihood algorithm combined with a χ^2^ test. Heterogeneity tests revealed a significantly different genotype distribution of SNPs 1, 11, 12, 17, 22 and 27 in both the Spanish and the German sample (data not shown). Overall, we detected differences between patients and controls for two SNPs (SNP14 and SNP27), which were most likely due to a specific association in the Spanish sample.

## Discussion

Here we present the results for a case-control association analysis that explores the impact of variation at *AHI1* locus on the vulnerability towards schizophrenia in two European samples of Caucasian origin from Southern Germany and East Spain. For this analysis, we focused on 29 tag SNPs located in the region corresponding to the linkage peak detected by Amann-Zalcenstein *et al.* [19]. Among these SNPs, we included those 7 SNPs which have been widely studied and associated with schizophrenia in different populations [19]–[21]. Several alleles and haplotypes were significantly associated with the risk for schizophrenia in the Spanish and, less pronounced, the combined sample. Some protective haplotypes were also found.

There are several reasons to consider *AHI1* a candidate gene for psychotic disorders. First, *AHI1* expression has been detected in brain areas particularly related to the pathophysiology of psychotic symptoms, including hippocampus, cerebral cortex, mesolimbic pathways, cerebellum, inferior colliculus and dorsal cochlear nucleus [15], [25]–[30]. Another interesting issue is the accelerated evolution, probably due to positive selection, of *AHI1* along the primate lineage leading to humans [15], [31]. Given that *AHI1* is critical for a proper neurodevelopment, accelerated evolution of *AHI1* in the human lineage may indicate the effect of directional selection. These observations suggest the involvement of this gene in the acquisition of higher cognitive and mental abilities in humans, a phenomenon which can be linked to the appearance of mental disorders such as schizophrenia [32]–[34]. Remarkably, it is not uncommon that genes causing monogenic rare diseases can also contribute to common complex diseases [35], [36]. Therefore, it cannot be excluded that more subtle variation in *AHI1* than those causing Joubert syndrome may have a role in the vulnerability to neuropsychiatric disorders.

While the study presented here was designed and performed, three case-control association studies reported the involvement of *AHI1* in the susceptibility to schizophrenia [19], [20] and autistic disorder [23]. According to these antecedents, we performed the present study, which intends to extend the study of *AHI1* gene to other populations in an attempt to find schizophrenia-associating markers in the Caucasian European context.

In summary, support for a role of *AHI1* in psychotic disorder was obtained in the sample from Valencia (East Spain), in which two SNPs located upstream from *AHI1* gene (SNP14-rs7750586 and SNP27-rs911507) were significantly associated with schizophrenia, with a moderate effect size (Odds Ratios (ORs) were 1.53 and 1.34, respectively). However, with regard to rs7750586, the risk allele associated with schizophrenia in our study (G allele) was different than in the two previous studies [19]–[20]. Moreover, we also found protective as well as risk haplotypes spanning SNP1 to SNP4, and SNP13 to SNP16, i.e. including the associated risk marker. This marker also impacted dimensional severity scores of schizophrenia, lending further support to the categorical case-control finding. Remarkably, many of the significant SNPs are located in putative regulatory regions. The significant haplotypes including SNP1 to SNP4 are of special interest because they span a region encoding the SH3 domain, one of the most important regions of Jouberin and particularly conserved in mammals [15], [16].

With regard to the negative results in the German sample, it should be noted that this sample was enriched for chronic, non-remitting schizophrenia, so that one might hypothesize that *AHI1* is associated with less severe, remitting forms of this disorder, a hypothesis which could easily be tested in further studies. In addition, we may also consider that the existence of differences in the clinical phenotyping of patients (DSM-IV criteria were used in the Spanish sample, whereas ICD-10 was the reference in the German dataset) may explain the differences observed between both samples in the case-control association analysis.

Interestingly, we also found some SNPs associating with schizophrenia in the combined German-Spanish sample. The higher size of this sample is of special interest because it increases the statistical power to detect association with the risk for schizophrenia (table 2). Indeed, sample size is one of the most important issues in the study of the genetics of complex diseases. However, the significant association observed in the joint dataset reduced significance of the results from the Spanish sample, therefore suggesting that the positive finding in the combined sample is mainly due to the SNP distribution in the Spanish sample. The differences among samples observed in the heterogeneity tests for some SNPs can also make it difficult to perform and interpret a study with a joint group of individuals from different origin. Indeed, it cannot be discarded that the differences observed between the Spanish and the German sample could be related to differences in the genetic structure of both populations. Although European populations share an important part of their genetic structure, they also present clear differences which should be taken into account when a case-control association analysis is designed [37]–[39]. These differences in the genetic structure of Spanish and German populations can involve other genetic interacting factors, which, in turn, may modulate the effect of the polymorphism of interest on the vulnerability to a complex disease. In addition, the differences in the directionality of the findings between the Spanish sample and previous reports also point to different issues. One paradigmatic example of this situation is the dysbindin gene, where every one of its five major haplotypes has been associated with schizophrenia [40]. This fact can be related, on one hand, to differences among populations and, on the other hand, to different study designs. In our study, to avoid an effect due to population stratification [41], [42], affected and control subjects were carefully matched on ethnicity, gender and age, and for the German sample the absence of population substructure has already been shown [43]. Finally, as it has been previously commented, we cannot discard that differences in the LD pattern may mask the existence of common causal variants among populations.

Finally, as a complement to our case-control association study, we evaluated the impact on several clinical variables (PANSS, BPRS and KGV scales) of those *AHI1* SNPs associated with schizophrenia in this study. Although we found a slight effect of *AHI1* gene on symptom severity, additional studies on more specific variables (particularly of cognitive nature) and with more individuals would be necessary to confirm this finding.

In conclusion, the findings from the present study lend support to the notion that the *AHI1* locus increases the genetic risk for psychoses at least in some populations. However, additional analyses with larger samples are necessary. Indeed, while this manuscript was in preparation, two additional analyses were published, one of them including a large number of different European samples [21], as well as another study that focused on a dense SNP map of the *AHI1* region [22]. Both studies also supported the hypothesis that *AHI1* is involved in the pathogenesis of schizophrenia. Therefore, our study, together with previous studies in different Caucasian populations, supports the relationship between genetic variation in *AHI1* and the risk for schizophrenia.

## Materials and Methods

### Ethics Statement

All subjects gave their written informed consent to participate in this study, which was approved by the Ethical Committees of the Medical Faculty, University of Valencia and the University of Würzburg.

### Clinical samples

The Spanish sample consisted of 280 unrelated psychotic patients and 348 control subjects. Patients came from the psychiatric in-patient and out-patient units of the Mental Health Service 4 of the Clinical Hospital, University of Valencia, Spain. Age ranged from 18 to 77 years. The retrospective clinical data collected from each patient were compared with the information provided from previous clinical reports and family members. All patients met DSM-IV criteria for different psychoses, mainly schizophrenia (76%). These diagnoses were confirmed for every patient by a consensus meeting with the treating psychiatrist and one of the psychiatrists of our research group. Patients also had a minimum one-year evolution of the illness and were on antipsychotic treatment at evaluation time. Exclusion criteria for this study included incoherence of speech and/or the incapacity for basic comprehension of the questions. Symptom severity was assessed in some patients through BPRS, KGV and PANSS scales (80, 200 and 80 individuals, respectively). The 348 healthy unrelated subjects had no history or familial background of psychiatric disorders. To avoid sample stratification, these subjects had similar demographic characteristics (Caucasian ethnic group, similar age) to the psychotic group. They were between 18 and 91 years old and were also of Spanish origin. Drug abuse was also considered among the exclusion criteria.

The German sample was enrolled at the Department of Psychiatry, Psychosomatics and Psychotherapy, University of Würzburg. A total of 247 unrelated patients meeting ICD-10 criteria for schizophrenia (mean age, 42 years) took part in the study. They were at least once in-patients at the Department of Psychiatry and none of the patients remitted completely during the course of disease, i.e. the sample was enriched for chronic and severe schizophrenia. Diagnoses were made by an extensive, semi structured interview performed by an experienced psychiatrist. Clinical data obtained from the interview was contrasted with the information obtained from family members and previous clinical assessments. Symptom severity was assessed in some individuals through BPRS. Patients did not show other disorders (neurological disorders, epilepsy, mental retardation) which could be the underlying cause of the psychiatric disorder. Substance-induced psychosis and affective disorders were also considered as exclusion criteria. A total of 360 control subjects were also included in the study. They were healthy blood donors from the same catchment area as the patient group (Lower Franconia, Bavaria). To avoid ethnic stratification, they were also from Caucasian origin. Although these individuals were not assessed for psychiatric disorders, all of them were free of medication and the aim of the study was explained to them, so it is unlikely that the subjects from the control group are suffering from severe psychiatric disorders. Only those patients and controls who gave their written informed consent were accepted for the study.

### Genotyping

**Figure 2:**
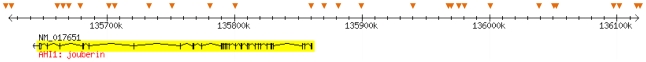
Genomic region and SNPs analyzed in this study. The position of each SNP is indicated with an orange arrow.

Genomic DNA was isolated from the peripheral blood of patients and controls according to standard procedures. Thirty single-nucleotide polymorphisms (SNPs) were initially selected from previous studies. However, one of them (rs6935033) was discarded during the design of the genotyping assay. Finally, twenty-nine SNPs were included in the study (see table 3 and figure 2 for more information about the markers). These SNPs covered the entire *AHI1* genomic region, as well as a 25 kb region located 5′ of *AHI1*. Upstream SNPs of *AHI1* were analyzed because the highest association signals from the original study from Amann-Zalcenstein *et al.* [19] were located in this region. SNP genotyping was performed in both samples through the iPLEX assay on the MassARRAY platform (Sequenom, San Diego, CA, USA), which allows high throughput genotyping through multiplex reactions. Exclusion criteria during quality control of the genotyping procedure were the following: HWE P-value below 0.05 in the control sample, genotyping rate below 70% and minor allele frequency (MAF) below 0.04. According to these criteria, one SNP (SNP18) was discarded in the Spanish sample (HWE P-value = 0.0389 in the control sample, genotyping rate 53,1%), as well as two SNPs (SNP7 and SNP19) were discarded in the German sample (genotyping rate 0% in the control sample). Moreover, in the Spanish sample, 25 controls and 37 patients were discarded due to the low genotyping rate, while two controls and three patients were discarded in the German sample. The high number of discarded samples in the Spanish dataset may be due to the DNA extraction procedures, given that a subset of Spanish DNA samples was extracted by the phenol-chloroform methodology.

**Table 3:** List of SNPs included in the present study.

rs code (or other name)	Position (dbSNP Build 127)	Marker order	Distance from SNP1	Allele change	CEU MAF	Location/Function
rs2327587	135622646	SNP1	0	C/T	0.408 (A)	3′ near gene
rs9494193	135625316	SNP2	2670	A/C	0.433 (C)	3′ near gene
rs4896142	135664512	SNP3	41866	G/T	0.383 (G)	Intron 25
rs6931735	135666504	SNP4	43858	A/G	0.408 (G)	Intron 25
rs6912933	135669227	SNP5	46581	A/G	0.425 (G)	Intron 25
rs9321501	135683110	SNP6	60464	A/C	0.397 (C)	Intron 24
rs2746429	135714977	SNP7	92331	C/T	0.395 (C)	Intron 23
rs2614258	135718895	SNP8	96249	A/G	0.350 (A)	Intron 23
rs2246943	135733209	SNP9	110563	A/T	0.400 (A)	Intron 22
rs2614264	135752387	SNP10	129741	A/G	0.400 (G)	Intron 22
rs11154801	135781048	SNP11	158402	A/C	0.317 (A)	Intron 19
rs2757643	135802926	SNP12	180280	A/C	0.381 (A)	Intron 13
rs6928455	135859828	SNP13	237182	C/T	0.051 (C)	Intron 2
rs7750586	135869366	SNP14	246720	A/G	0.325 (G)	5′ near gene
rs9647635	135882749	SNP15	260103	A/C	0.322 (C)	5′ near gene
rs12203875	135902107	SNP16	279461	A/G	0.383 (G)	5′ near gene
rs10484771	135939628	SNP17	316982	C/T	0.058 (T)	5′ near gene
rs4896156	135968556	SNP18	345910	A/G	0.322 (G)	5′ near gene
rs9376112	135969693	SNP19	347047	A/C	0.308 (C)	5′ near gene
rs2143681	135974698	SNP20	352052	A/G	0.314 (A)	5′ near gene
rs6925684	135978571	SNP21	355925	A/G	0.312 (A)	5′ near gene
rs6902485	135999485	SNP22	376839	A/G	0.042 (G)	5′ near gene
rs7739635	136039471	SNP23	416825	C/T	0.322 (T)	5′ near gene
rs9494332	136050301	SNP24	427655	A/G	0.280 (G)	5′ near gene
rs2273069	136052491	SNP25	429845	A/G	0.310 (G) in Asians	5′ near gene
rs1475069	136097927	SNP26	475281	A/C	0.283 (C)	5′ near gene
rs911507	136101116	SNP27	478470	A/G	0.230 (G)	5′ near gene
rs12211505	136116514	SNP28	493868	C/T	0.250 (C)	5′ near gene
rs2208574	136117310	SNP29	494664	A/G	0.258 (A)	5′ near gene

Abbreviations: CEU, CEPH collection - DNA samples of Utah residents with ancestry from northern and western Europe; MAF, minor allele frequency.

### Statistical analysis

Prior to any association analysis, it is important to make a priori calculation to know the statistical power of the study. This power varies attending to different parameters, such as the sample size, the risk allele frequency, the incidence of the disease and the risk (odds ratio) assumed for each polymorphism, among others. For this study, QUANTO software version 1.2.3 was used to calculate the statistical power to find associations between genetic polymorphisms and the risk for schizophrenia. We assumed an odds ratio (OR) between 1.3 and 2. We also set the incidence of schizophrenia at 1% and the inheritance model as additive. The estimations for our different samples are summarized in table 4. Particularly, this power ranged between 0.149 and 0.999 depending on the sample size, the risk associated to each allele and the allele frequencies. Thus, for those polymorphisms with a low MAF, the statistical power to detect association with the disease is expected to be lower, especially if the risk is low. In our particular case, 21 of the 29 SNPs presented a MAF equal to or higher that 0.3, and only 3 SNPs had a MAF lower than 0.1. Therefore, we consider the statistical power of the study to be adequate.

**Table 4:** Estimation of the statistical power associated to the different case-control and family-based studies.

Type of study	Sample	N	Frequency of the risk allele^a^	Statistical Power		
				OR = 1.3	OR = 1.6	OR = 1.9
Case-control	Spanish	243 patients	0.04	0.149	0.307	0.678
		323 controls	0.09	0.261	0.554	0.933
			0.14	0.355	0.709	0.984
			0.19	0.429	0.801	0.995
			0.24	0.487	0.855	0.998
			0.29	0.529	0.887	0.999
			0.34	0.558	0.906	0.999
			0.39	0.577	0.916	0.999
			0.44	0.586	0.919	0.999
Case-control	German	243 patients	0.04	0.153	0.318	0.698
		358 controls	0.09	0.271	0.573	0.943
			0.14	0.368	0.728	0.987
			0.19	0.445	0.818	0.997
			0.24	0.503	0.870	0.999
			0.29	0.546	0.899	0.999
			0.34	0.576	0.917	0.999
			0.39	0.594	0.926	0.999
			0.44	0.603	0.929	0.999
Case-control	Combined	486 patients	0.04	0.252	0.540	0.930
		681 controls	0.09	0.464	0.843	0.978
			0.14	0.613	0.945	0.989
			0.19	0.713	0.978	0.999
			0.24	0.779	0.989	0.999
			0.29	0.821	0.994	0.999
			0.34	0.847	0.996	0.999
			0.39	0.862	0.997	0.999
			0.44	0.869	0.997	0.999

**^a^**The risk allele was considered to be the minor allele.

Abbreviation: OR, Odds Ratio.

Genotypes were assessed for Hardy-Weinberg Equilibrium (HWE) in both patient and control samples by applying a χ^2^ test implemented with Haploview Program version 4 [44]. As the deviation from HWE in the control sample could be indicating a genotyping error, those SNPs which deviated from HWE in the control sample (P<0.1) were discarded from the analysis.

Differences in the allelic frequencies between patients and controls were evaluated with a χ^2^ test via Unphased program version 3.0.12 [45], [46]. Moreover, the web-based application SNPStats [47] was used to compare, by means of logistic regression, the genotypic frequencies between groups. Different inheritance models (codominant, dominant, recessive, and additive) were tested. ORs and 95% confidence intervals as indicators of the risk associated to each genotype were also calculated. Finally, if any SNP was found to be associated with the risk for schizophrenia, a linear regression analysis with SNPstats was used to evaluate the effect of those SNPs on several clinical scores (BPRS, PANSS and KGV). Bonferroni sequential test for multiple comparisons [48] was applied to correct all the reported p-values.

Regarding the haplotype analysis, haploblock frequencies were compared between patients and controls with Haploview Program version 4 [44]. Moreover, frequencies of sliding windows of two, three and four-marker haplotypes were estimated through a retrospective likelihood algorithm and compared between patients and controls with the Unphased software version 3.0.12 [45], [46]. It should be noted that haplotypes with a frequency below 0.03 were considered as rare and therefore discarded from the statistical analysis. A 1000-permutation run was performed in all haplotype analyses to better estimate the significance of the positive associations.

We also performed a joint analysis by combining the affected individuals and healthy subjects from Spain and Germany. The linkage disequilibrium patterns between SNPs were determined to assess the validity of such type of analysis. Moreover, these analyses were only stratified according to the region of origin of each individual (Germany or Spain) since there were no differences in other possible stratification variables, for example ethnicity. PLINK software version 1.0.2 [49] was used to apply two different CMH tests, which are based on an “average” OR that controls for the potential confounding due to the cluster variable. Two variants of the CMH tests were performed: a) a CMH test that performs the case-control association test but controlling the effect of the cluster variable (in our particular case, the cluster variable was the country of origin); b) a CMH test that is used as a measure of the heterogeneity between samples, which assesses whether a particular SNP varies between clusters. Finally, we also performed a haplotype analysis following the same procedures described above for the Spanish and the German sample separately.
